# Supplementary material for: Bridging Gaps in Obesity Assessment: Spanish Validation of the Eating Behaviors Assessment for Obesity (EBA-O)
Source: Nutrients. 2025 Jul 17;17(14):2344. doi: 10.3390/nu17142344 (PMC12300351; doi:10.3390/nu17142344)
Supplement: Supplementary file 1 [file nutrients-17-02344-s001.zip › nutrients-3759236-supplementary.pdf]

nutrients

MDPI

Table S1. Spanish version of the EATING BEHAVIOR ASSESSMENT FOR OBESITY (EBA-O)

EATING BEHAVIORS ASSESSMENT FOR OBESITY (EBA-O)

EN LOS ULTIMOS 3 MESES	Nunca	Menos de 1 vez al mes	1 vez al mes	2-3 veces al mes	1 vez a la semana	2-3 veces a la semana	4-6 veces a la semana	Cada día
1. ¿Alguna vez te has despertado en mitad de la noche y has sentido el deseo de comer algo?	○	○	○	○	○	○	○	○
2. En caso de despertarte por la noche, ¿has sido incapaz de dormir sin comer algo?	○	○	○	○	○	○	○	○
3. ¿Alguna vez has comido algo después de la cena para dormir mejor?	○	○	○	○	○	○	○	○
4. ¿Alguna vez no has podido dormir sino comías antes de acostarte, incluso después de haber cenado normalmente?	○	○	○	○	○	○	○	○
5. ¿Alguna vez has tenido el impulso de comer ciertos alimentos altos en carbohidratos/grasas y no poder pensar en otra cosa?	○	○	○	○	○	○	○	○
6. Cuando has sentido ese impulso, ¿hiciste todo lo posible por conseguir estos alimentos, a pesar de tener otros alimentos disponibles?	○	○	○	○	○	○	○	○
7. ¿Te ha pasado que no podías parar de comer estos alimentos a pesar de saber que eran dañinos para tu salud?	○	○	○	○	○	○	○	○
8. ¿Has intentado reducir o dejar de comer estos alimentos sin conseguirlo?	○	○	○	○	○	○	○	○
9. ¿Te has sentido irritable, nervioso/a, triste o has tenido síntomas físicos (ej. dolor de cabeza, cansancio/fatiga) cuando has reducido o dejado de comer estos alimentos y has tenido que comerlos para sentirte mejor?	○	○	○	○	○	○	○	○
10. ¿Has tenido muchas ganas de comer dulces?	○	○	○	○	○	○	○	○
11. ¿Has consumido a menudo alimentos dulces y/o bebidas azucaradas?	○	○	○	○	○	○	○	○
12. ¿Has tenido muchas ganas de comer dulces o beber bebidas azucaradas cuando estabas ansioso/a o triste?	○	○	○	○	○	○	○	○
13.En comparación con otras personas, ¿tus porciones de alimentos eran mucho más grandes?	○	○	○	○	○	○	○	○
14. ¿Has repetido algún plato durante las comidas principales?	○	○	○	○	○	○	○	○

15. ¿Has comido demasiado, pero sólo en las comidas principales?	○	○	○	○	○	○	○	○
16. ¿has comido rápidamente, en un cierto período de tiempo, hasta sentirte incómodamente lleno/a, mucha más comida de la que otras personas comerían en el mismo tiempo y en circunstancias similares?	○	○	○	○	○	○	○	○
17. De ser así, ¿tuviste la sensación de perder el control y no poder dominar lo que comías, cuánto comías y cuándo parar de comer?	○	○	○	○	○	○	○	○
18. ¿Has sentido vergüenza o culpa por este comportamiento?	○	○	○	○	○	○	○	○

Table S2. Fit indices for measurement invariance tests for sex.

Robust model fit indices							Model difference						
Model	χ^2	df	CFI	TLI	RMSEA	SRMR	ΔM	$\Delta\chi^2$	Δdf	ΔCFI	ΔTLI	ΔRMSEA	ΔSRMR
M1	673.00	260	0.98	0.98	0.07	0.08							
M2	691.80	277	0.98	0.97	0.08	0.09	M2 VS. M1	18.801	17	0.001	0.003	-0.005	-0.001
M3	716.04	378	0.98	0.98	0.07	0.09	M3 VS. M2	24.243	12	0.000	-0.001	-0.004	0.000

M1: configural invariance; M2: metric invariance; M3: scalar invariance. All the $\Delta\chi^2$ were not significant.

Table S3. Fit indices for measurement invariance tests for BMI.

Robust model fit indices							Model difference						
Model	χ^2	df	CFI	TLI	RMSEA	SRMR	ΔM	$\Delta\chi^2$	Δdf	ΔCFI	ΔTLI	ΔRMSEA	ΔSRMR
M1	707.98	260	0.93	0.91	0.07	0.08							
M2	729.69	277	0.92	0.91	0.08	0.09	M2 VS. M1	21.706	17	0.001	0.000	-0.001	-0.001
M3	768.56	378	0.92	0.90	0.07	0.08	M3 VS. M2	38.872	12	-0.002	0.001	0.001	0.001

M1: configural invariance; M2: metric invariance; M3: scalar invariance. All the $\Delta\chi^2$ were not significant.
